# Supplementary material for: Causal Relationship between Aging and Anorexia Nervosa: A White-Matter-Microstructure-Mediated Mendelian Randomization Analysis
Source: Biomedicines. 2024 Aug 16;12(8):1874. doi: 10.3390/biomedicines12081874 (PMC11351342; doi:10.3390/biomedicines12081874)
Supplement: Supplementary file 1 [file biomedicines-12-01874-s001.zip › Supplementary Figure/Figure S9/Leaveoneout plot of MR analysis for ubm-b-1523 to AN.pdf]

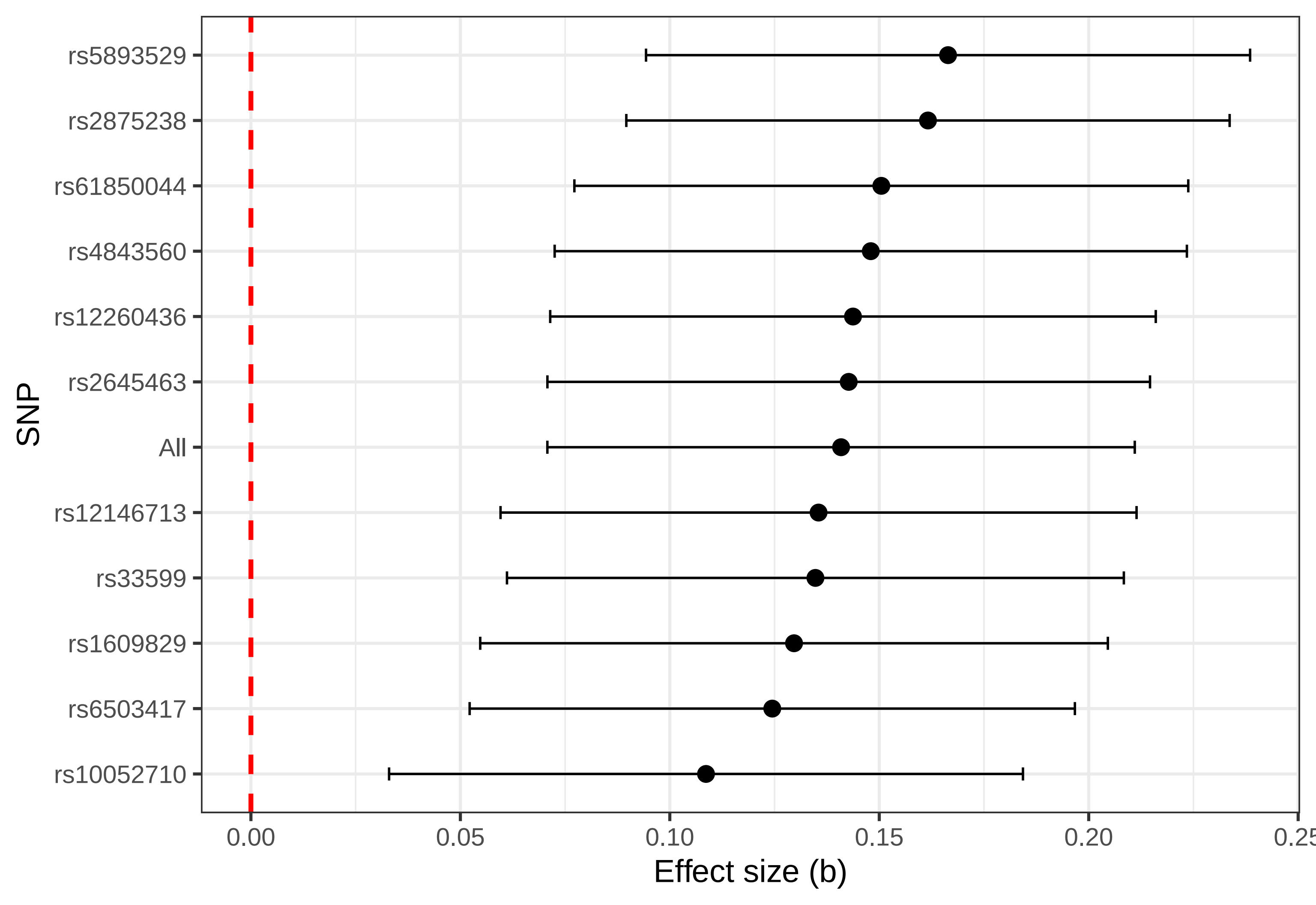
rs5893529
rs2875238
rs61850044
rs4843560
rs12260436
rs2645463
All
rs12146713
rs33599
rs1609829
rs6503417
rs10052710
SNP
0.00
0.05
0.10
0.15
0.20
0.25
Effect size (b)
